# Restoration of H3k27me3 Modification Epigenetically Silences Cry1 Expression and Sensitizes Leptin Signaling to Reduce Obesity‐Related Properties

**DOI:** 10.1002/advs.202004319

**Published:** 2021-05-13

**Authors:** Yan Wei, Jun Chen, Xing Xu, Fan Li, Kun Wu, Yingying Jiang, Yuqing Rao, Chen Zhao, Wantao Chen, Xu Wang

**Affiliations:** ^1^ Department of Ophthalmology and Vision Science Eye and ENT Hospital, Shanghai Medical College NHC Key Laboratory of Myopia Fudan University Shanghai 200031 China; ^2^ Department of Oral and Maxillofacial‐Head and Neck Oncology Shanghai Ninth People's Hospital Shanghai Jiao Tong University School of Medicine College of Stomatology, Shanghai Jiao Tong University National Center for Stomatology National Clinical Research Center for Oral Diseases Shanghai 200011 China; ^3^ Shanghai Key Laboratory of Stomatology and Shanghai Research Institute of Stomatology Shanghai 200011 China; ^4^ Department of Ophthalmology Shanghai Xinhua Hospital Shanghai Jiao Tong University School of Medicine Shanghai 200092 China

**Keywords:** Cry1, hypothalamus, Kdm6a, leptin, obesity

## Abstract

The trimethylation on histone H3 lysine 27 (H3k27me3), a transcriptionally repressive epigenetic mark of permissive chromatin, can be removed by the histone lysine demethylase 6a (Kdm6a). However, the physiological function of H3k27me3 and Kdm6a on circadian genes remains largely elusive. With the ChIP‐Seq and mRNA microarray assays, a critical role is identified for Kdm6a in the regulation of H3k27me3 to impact the expression of Crytochrome 1 (Cry1) in the hypothalamus of diet induced obesity mice. More importantly, both conditional knockout and pharmacological inhibition of *Kdm6a* reduce body weight and stabilize blood glucose homeostasis. Although a Kdm6a inhibitor fails to decrease body weight in leptin receptor‐deficient db/db mice, it significantly decreases Cry1 expression, enhances sensitivity to exogenous leptin administration, and blocks body weight increases in endo‐leptin‐deficient ob/ob mice. Moreover, gene analysis of the human hypothalamus further reveals a positive correlation between Kdm6a and Cry1. The results show that inhibition of Kdm6a reduces the Cry1 expression and sensitizes leptin signaling to combat obesity‐related disease. Therefore, it implicates Kdm6a as an attractive drug target for obesity and metabolic disorders.

## Graphical abstract

Both conditional knockout and pharmacological inhibition of Kdm6a reduce body weight and stabilize blood glucose homeostasis. Inhibition of Kdm6a restores the H3k27me3 modification, reduces the Cry1 expression in the hypothalamus, and sensitizes leptin signaling to combat obesity‐related disease. Therefore, it implicates Kdm6a as an attractive drug target for obesity and metabolic disorders.

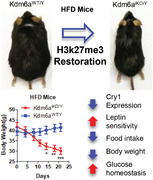

## 1 Introduction

The trimethylation on histone H3 lysine 27 (H3k27me3) is a histone modification occurring on the amino terminal tail of the core histone H3. This trimethylation is associated with the downregulation of nearby genes via the formation of heterochromatic regions.^[^ ^1^ ^]^ It acts in opposition to the trimethylation on histone H3 lysine 4 (H3k4me3), which activates gene expression. Because of its dramatic and predictable effect on gene expression, H3k27me3 is a favorite of epigenetic researchers looking for inactive genes. H3k27me3 can be removed by the histone lysine demethylases 6 (Kdm6). The Kdm6 family contains a Jmjc‐domain^[^ ^2^ ^]^ and counters the enzymatic activity of polycomb repressive complex 2^[^ ^3^ ^]^ by removing di‐ and trimethyl groups from histone H3 lysine 27 (H3k27). The Kdm6 family includes Kdm6a (also known as Utx) and Kdm6b (also known as Jmjd3) and has been shown to play important roles in a multitude of cellular processes, including differentiation,^[^ ^4^, ^5^ ^]^ senescence,^[^ ^6^ ^]^ somatic and germ cell reprogramming,^[^ ^7^ ^]^ the inflammatory response,^[^ ^8^ ^]^ and cancer.^[^ ^9^ ^]^ The discovery of GSK‐J4 provides an effective tool for the pharmacological inhibition of Kdm6 members, which have been reported to inhibit the growth of TAL‐1 positive T‐acute lymphoblastic leukemia,^[^ ^10^, ^11^ ^]^ diffuse intrinsic pontine glioma,^[^ ^12^, ^13^ ^]^ melanoma,^[^ ^14^ ^]^ and neuroblastoma.^[^ ^15^ ^]^ Most recently, Kdm6a deficiency or inhibition of Kdm6 members with GSK‐J4 abated nephropathy progression in diabetic db/db mice, suggesting that Kdm6a is also involved in the progression of metabolic diseases.^[^ ^16^ ^]^ Although Kdm6a has been reported as one of the targets of the well‐known antidiabetic drug metformin,^[^ ^17^ ^]^ it is not fully understood what the physiological role of Kdm6a is in metabolic diseases, such as obesity and diabetes. In particular, the relationship between Kdm6a and leptin resistance has not been investigated in the obesity.

The circadian clock genes primarily comprise transcriptional/translational feedback loops involving a set of clock genes, to adapt daily 24 h changes in the environment and enable mammals to maintain physiological homeostasis.^[^ ^18^ ^]^ The cryptochrome 1 (Cry1) is part of the negative regulatory arm of the circadian clock system as the rhythmical accumulation leads to the formation of a repressor complex that interacts with circadian locomotor output cycles kaput (Clock) to inhibit the transcription.^[^ ^19^ ^]^ The perturbations of endoplasmic reticulum (ER) homeostasis that are triggered predominantly by the accumulation of unfolded proteins lead to the development of a condition referred to as ER stress.^[^ ^20^ ^]^ In the hypothalamus, ER stress can induce circadian disruption and linking leptin resistance with obesity,^[^ ^21^ ^]^ implicating the central clock in body weight control and the development of metabolic abnormalities. Mice deficient in Cry1^−/−^ exhibit resistance to the high fat diet (HFD) induced obesity, indicating Cry1 is a plausible target for antiobesity therapy.^[^ ^22^ ^]^ However, the H3k27me3‐based regulation on the Cry1 expression has not been well illustrated.

Leptin is an important adipocyte‐derived hormone and the first‐identified messenger to carry peripheral information about energy cues to the central nervous system.^[^ ^23^, ^24^ ^]^ However, initial hopes for leveraging the anorectic effect of leptin for treating obesity quickly diminished, as the hormone is ineffective in creating satiety and suppressing food intake, despite the presence of high levels of circulating leptin in murine models of obesity and in obese humans. Furthermore, while exogenously administered leptin doses suppress food intake and reduce body weight in lean mice, this approach is ineffective in diet‐induced obese (DIO) mice. These findings led to the notion that obesity is a pathological condition associated with leptin resistance. Despite long‐standing research efforts, agents that can alleviate leptin resistance have rarely been found.

In this study, we used an inducible knockout model and the Kdm6a inhibitor GSK‐J4 to examine the influence of Kdm6a and H3k27me3 on Cry1 expression in the obese hypothalamus and leptin sensitivity in obese mice. We verified that Kdm6a inhibition restored the H3k27me3 modification and reduced appetite, which is largely dependent on the leptin‐signaling pathway.

## 2 Results

### 2.1 Deficiency of Kdm6a Attenuates Body Weight in DIO Mice

**Figure 1:**
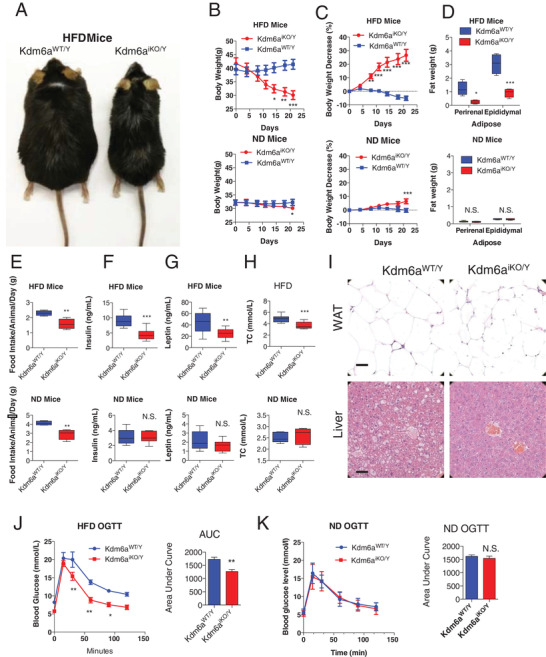
The inducible *Kdm6a* knockout reduces the body weight and food intake of DIO mice but not lean mice. A) Representative images of wild‐type or knockout mice fed with HFD. B,C) Wild‐type mice and Kdm6a‐inducible deficient mice were observed for 21 d. Body weight and percentage change in body weight (*n* = 10 pre group). D) Fat weights were recorded. E) Daily food intake during the second week of treatment. F) Serum insulin concentrations of DIO or lean mice after 3 weeks of inducible knock out treatment. G) Serum leptin concentrations of DIO or lean mice after 3 weeks of treatment. H) The total cholesterol (TC) concentration in the serum of the mice was examined after 3 weeks of treatment. ***p < 0.001; **p < 0.005; *p < 0.05; N.S., not significant. I) The representative images of hematoxylin eosin (HE)‐stained epididymal white adipose tissue (WAT) and liver of wild‐type or Kdm6a‐deficient mice fed an HFD, bar = 100 µm, *n* = 3 mice per group. J) OGTT assay to detect blood glucose homeostasis in DIO mice. K) OGTT assay to detect blood glucose homeostasis in lean mice.

We previously found that the H3k27me3 modification decreased under ER stress status (Figures S1A–N and S2A–J, Supporting Information). Given that ER stress has been linked to obesity in vivo,^[^ ^25^ ^]^ to further examine the potential physiological effect of Kdm6a in vivo, we established inducible knockout mice and selected a high‐fat diet‐induced obesity model. The genotypes of the *Kdm6a^F/Y^; Cre/ERT2^+^* mice were identified by a polymerase chain reaction (PCR) assay (Figure S3A, Supporting Information). Mice with validated genotypes were administered tamoxifen intraperitoneally (i.p.) daily for 4 d, leading to the functional activation of Cre/ERT2 to knock out the *Kdm6a* gene (Figure S3B,C, Supporting Information). Notably, the inducible knockout of *Kdm6a* significantly reduced the body weights of DIO mice (**Figure** **1**A). Under HFD‐fed conditions, after tamoxifen administration, the body weights of the Kdm6a^iKO/Y^ mice were nearly 30% lower than those of the mice before treatment (Figure 1B,C). Consistent with this finding, the *Kdm6a* knockout exhibited significantly less perirenal and epididymal adipose tissue than its wild‐type counterparts fed with HFD (Figure 1D). We also examined the influence of the *Kdm6a* knockout on the body weights of mice fed a normal diet (ND). Notably, after treatment with tamoxifen at the same dosage as the HFD group, the body weights of Kdm6a^iKO/Y^ mice fed with ND were comparable to those of the wild‐type group.

As the decline in food intake is the major cause of body weight reduction, the average food intake was measured. *Kdm6a^iKO/Y^* mice consumed significantly less food than the *Kdm6a* ^WT/Y^ group of DIO mice (Figure 1E). We also found that the concentrations of insulin and leptin in the *Kdm6a^iKO/Y^* mice were much lower than those in the *Kdm6a^WT/Y^* mice (Figure 1F,G). *Kdm6a* knockout obviously enhanced the metabolic status of DIO mice (Figure 1H) and decreased the adipocyte size in the epididymal fat (Figure 1I). Moreover, oral glucose tolerance test (OGTT) assays revealed that the inducible knockout of *Kdm6a* selectively enhanced glucose homeostasis in obese mice (Figure 1J) but not in lean mice (Figure 1K). Although the food intake of the mice fed with ND was obviously reduced by the *Kdm6a* knockout in week 1, there were no significant changes in insulin, leptin levels compared to those in the wild‐type group at the end of week 3. The insulin tolerance test (ITT) assay data indicate that the Kdm6a‐deficiency could not obviously change the sensitivity to insulin, neither in the HFD mice nor the ND mice (Figure S3D,E, Supporting Information).

**Figure 2:**
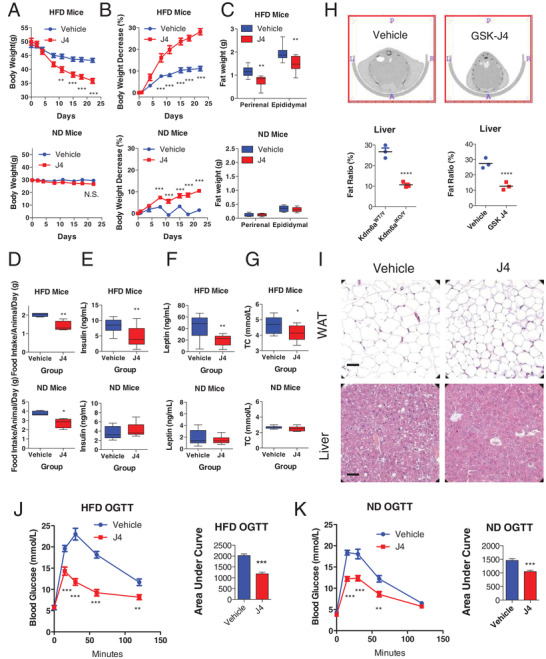
The Kdm6a inhibitor GSK‐J4 reduces the body weight and food intake of DIO mice but not lean mice. A,B) Mice with DIO received vehicle or the Kdm6a inhibitor GSK‐J4 (30 mg kg^−1^) for 21 d. Body weight and percentage change in body weight (for HFD mice, vehicle, *n* = 10; GSK‐J4, *n* = 9; for ND mice, vehicle, *n* = 10; GSK‐J4, *n* = 10). C) Fat weights were recorded. D) Daily food intake during the second week of treatment. E) Serum insulin concentrations of mice after 3 weeks of treatment. F) Serum leptin concentrations of mice after 3 weeks of treatment. G) Serum total cholesterol concentrations of mice after 3 weeks of treatment. ***p < 0.001; **p < 0.005; *p < 0.05. N.S., not significant. H) Representative cross‐sectional images of wild‐type and Kdm6a‐deficient mice subjected to microcomputed tomography analysis of the in situ accumulation of fat. Hepatic fat content was calculated in the genetically modulated mice and pharmacologically treated mice (*n* = 3 mice per group). All data represent the mean ± s.e.m. ****p < 0.0001 versus. I) The representative images of HE‐stained epididymal WAT and liver of vehicle‐ or GSK‐J4‐treated mice fed with HFD, bar = 100 µm, *n* = 3 mice per group. J) OGTT assay to detect blood glucose homeostasis in DIO mice. K) OGTT assay to detect blood glucose homeostasis in lean mice.

To investigate whether pharmacological inhibition of Kdm6a with GSK‐J4 can phenocopy the genetic ablation of *Kdm6a* by exerting an antiobesity effect, we administered GSK‐J4 to DIO and lean mice (Figure S4A, Supporting Information). As illustrated in **Figure** **2**A,B, the administration of GSK‐J4 reduced the body weight from 49.99 ± 1.23 g on day 1 to 35.85 ± 0.87 g on day 22 (*p* < 0.001), corresponding to a 28.15% ± 1.45% decrease relative to the initial body weight. The adipose tissue and food intake of the GSK‐J4‐treated DIO mice was also significantly reduced compared to that of the vehicle‐treated group (Figure 2C,D). Although the administration of GSK‐J4 to lean mice cause a reduction of food intake, the treatment did not induce a significant change in body weight compared to these parameters in the respective control groups (Figure 2A,B). The GSK‐J4 treatment clearly reduced fasting blood glucose during the 3‐week administration period (Figure S4B, Supporting Information), while the glucose level of lean mice did not change significantly under this experimental condition (Table S1, Supporting Information). The concentrations of insulin and leptin in the GSK‐J4‐treated mice were much lower than those in the vehicle‐treated mice (Figure 2E,F). The GSK‐J4 treatment obviously enhanced the metabolic status of DIO mice (Figure 2G). Microcomputed tomography scanning revealed that 16‐week‐old *Kdm6a*‐deficient mice or GSK‐J4 treated mice stored much less fat than the control mice (Figure 2H). The adipocyte size and liver steatosis were markedly decreased following the inhibition of Kdm6a functions by pharmacological inhibition (Figure 2I). OGTT assays revealed that the GSK‐J4 enhanced glucose homeostasis in the mice (Figure 2J,K). The responses of both the obese and lean mice to insulin stimulation were significantly affected, as shown in the ITT assay (Figure S4C,D, Supporting Information).

**Figure 3:**
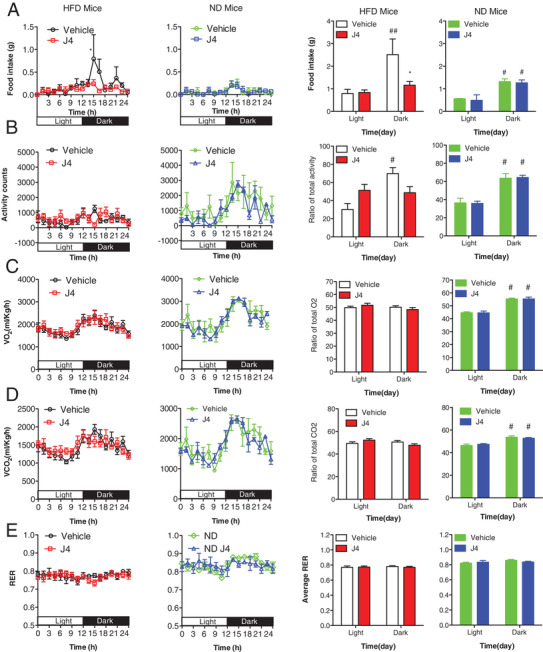
The pharmacological inhibition on Kdm6a decreases food intake of DIO mice but not lean mice. A) The mice received vehicle or GSK‐J4 (30 mg kg^−1^) for 10 d. The food intake was recorded. *p < 0.05, GSK‐J4 vs Vehicle; #*p* < 0.05, ##*p* < 0.01, light vs dark phage (*n* = 5 in each group of HFD mice; *n* = 3 in each group of ND mice for (A)–(E)). B) The locomotor activities of mice. #*p* < 0.05, light vs dark phage. C) Oxygen consumption (VO2) monitored over a 24 h period, shown as averaged values. #*p* < 0.05, light vs dark phage. D) Carbon dioxide production (VCO2) monitored over a 24 h period, shown as averaged values. #*p* < 0.05, light vs dark phage. E) The respiratory exchange rate (RER) was analyzed for 24 h. #*p* < 0.05, light vs dark phage.

Next, we examined the daily activity and food intake of mice treated with Kdm6a inhibitor GSK‐J4. The DIO mice with GSK‐J4 administration consumed much less portion of food intake during dark phase and more portions during light phase compared to the vehicle group (**Figure** **3**A). But there were no differences between the GSK‐J4 and Vehicle group in the normal diet mice (Figure 3B). The GSK‐J4 treatment changed the locomotor activity in the DIO mice (Figure 3B), resulting to the nearly equal radio of light and dark phage. In both DIO and lean mice, the GSK‐J4 treatment did not changed the oxygen consumption and carbon dioxide production, compared to the vehicle group (Figure 3C,D). In the meanwhile, there is no obvious different respiratory exchange rate (RER) between Vehicle‐ and GSK‐J4‐treated mice (Figure 3E). These data suggest that GSK‐J4 treatment selectively changed the daily food intake and activity of DIO mice.

Taken together, these data indicated that both genetically deficiency and pharmacological inhibition of Kdm6a selectively attenuated body weight and improved glucose homeostasis in DIO mice.

### 2.2 H3k27me3 Restoration Silences Cry1 Gene Expression in the Hypothalamus

**Figure 4:**
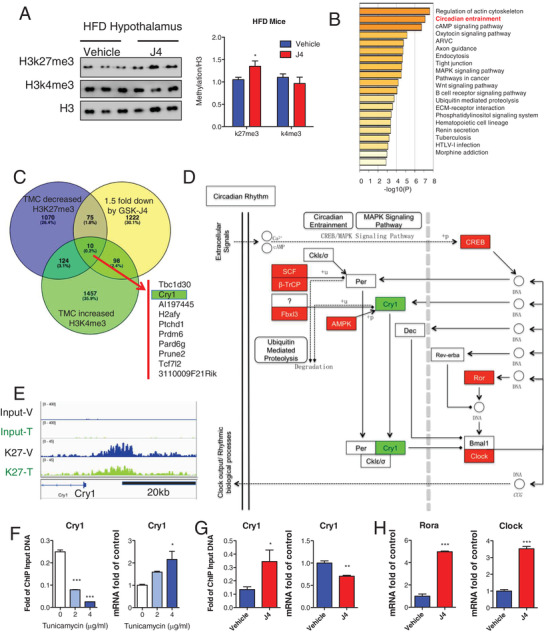
Kdm6a epigenetically controls Cry1 expression through H3k27me3 modification. A) Immunoblotting images of H3k27me3 and H3k4me3 in the hypothalamus of DIO mice treated with vehicle or 30 mg kg^−1^ GSK‐J4. B) The enrichment of KEGG pathway indicates the circadian entrainment gain high score in the hypothalamus of GSK‐J4‐treated mice compared to the vehicle group of mice. C) Venn diagram showing the numbers of genes harboring decreased H3k27me3 peaks, increased H3k4me3 peaks in tunicamycin treated MEFs and reduced mRNA expression in GSK‐J4 treated hypothalamus. D) The KEGG enrichment of over 1.5‐fold changed circadian genes is based on the analysis of GSK‐J4 treated hypothalamus. E) Snapshot of H3k27me3 ChIP‐Seq signal at the *Cry1* loci in vehicle and tunicamycin treated MEFs. F) ChIP‐qPCR analysis of H3k27me3 modifications at the *Cry1* loci in vehicle and tunicamycin treated MEFs as well as the real‐time PCR analysis of *Cry1* mRNA (*n* = 3). G) ChIP‐qPCR analysis of H3k27me3 modifications at the *Cry1* loci in vehicle and GSK‐J4 treated hypothalamus of DIO mice as well as the real‐time PCR analysis of *Cry1* mRNA (*n* = 3). H) Real‐time PCR analysis of other circadian genes Rora and Clock in the hypothalamus of DIO mice (*n* = 3).

As the hypothalamus is the key region of the brain that mediates ER stress signals, the hypothalamic tissues of DIO mice were collected after the treatment of Kdm6a inhibitor GSK‐J4. The immunoblotting assay data indicated that the Kdm6a inhibitor increased the H3k27me3 modification in the hypothalamus (**Figure** **4**A). A microarray analysis was further performed to determine the gene expression profile in the hypothalamus of DIO mice. The significantly 1.5‐fold changed genes were enriched by using terms from the kyoto encyclopedia of genes and genomes (KEGG) pathway (Figure 4B and Figure S2I,J, Supporting Information) and the circadian entrainment pathway is one of the most top enriched pathways (Figure S5, Supporting Information).

To correlate chromatin binding in tunicamycin‐induced ER stress with direct gene regulations in GSK‐J4 treated hypothalamus, we integrated the H3k27me3‐ and H3k4me3‐dependent histone modification with a primary focus on the genes displaying down‐regulation of hypothalamus. There are only ten genes with reduced H3k27me3 modification and increased H3k4me3 modification under the tunicamycin‐induced ER stress with previous ChIP‐Seq assay. Moreover these ten genes are also downregulated by GSK‐J4 administration in the hypothalamus of DIO mice (Figure 4C). Among these H3k27me3 directly regulated genes, we noticed the circadian gene Cry1. We further analyzed the transcriptome of GSK‐J4 treated hypothalamus and found that Cry1 is downregulated as well its target gene Clock was upregulated (Figure 4D). Interestingly, we identified Cry1 harbored several H3k27me3 intervals near the transcription start site (Figure 4E). The tunicamycin treatment reduced occupancies of H3k27me3 in the *Cry1* locus by ChIP‐quantitative polymerase chain reaction (qPCR) assays and reactivated expression of *Cry1* mRNA (Figure 4F). GSK‐J4 administration also epigenetically reduced the mRNA level of *Cry1* and increased H3k27me3 modification levels on *Cry1* promoter in the hypothalamus of DIO mice (Figure 4G). We also validated that the expression of circadian genes of Clock and Rora, the target gene of Cry1, were significantly increased by GSK‐J4 treatment in DIO mice (Figure 4H). These data suggest that the reversed H3k27me3 modification could reduce Cry1 in the hypothalamus of DIO mice.

### 2.3 Kdm6a Inhibition Reduces Cry1 Expression and Improves Leptin Sensitivity in the Hypothalamus of Mice

**Figure 5:**
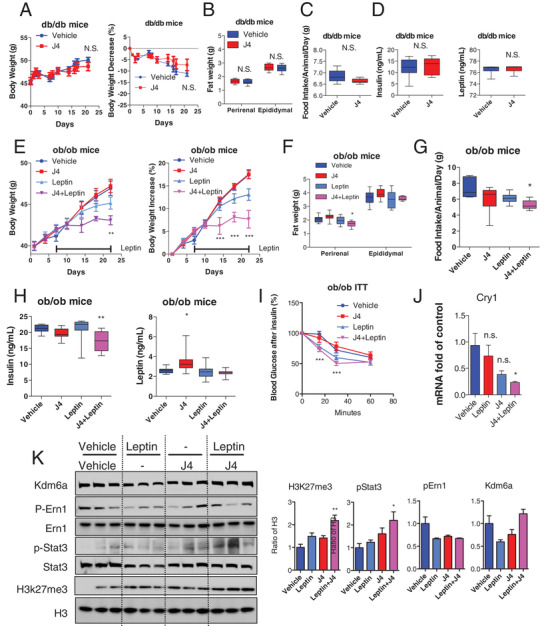
The Kdm6a inhibitor GSK‐J4 acts as a leptin sensitizer in mouse models to modulate downstream leptin signaling. A) The db/db mice were injected i.p. daily with vehicle or GSK‐J4 (30 mg kg^−1^) for 3 weeks. The body weights of obese mice are indicated (*n* = 10 for each group). The percent decrease (%) in body weight of obese mice was calculated during the treatment. B) The fat weight of perirenal and epididymal adipose tissues was collected after the treatment (*n* = 10 for each group). C) The average food intake of obese mice was recorded during the second week of treatment (*n* = 10 for each group). D) The insulin and leptin concentration in the serum of obese mice was examined after 3 weeks of treatment (*n* = 10 for each group). E) The ob/ob mice were injected i.p. daily with vehicle or GSK‐J4 (30 mg kg^−1^) in the presence or absence of leptin for 3 weeks. The body weights of obese mice are indicated (*n* = 10 for each group). The percent decrease (%) in body weight of obese mice was calculated during the treatment. F) The fat weight of perirenal and epididymal adipose tissues was collected after the treatment (*n* = 10 for each group). G) The average food intake of each group of mice was recorded during the second week of treatment (*n* = 10 for each group). H) The insulin and leptin concentration of the mice was examined after 3 weeks of treatment (*n* = 10 for each group). ***p < 0.001; **p < 0.005; *p < 0.05. N.S., not significant. I) ITT assay to detect blood glucose homeostasis in ob/ob mice. ***p < 0.001. J) Real‐time PCR assay to show *Cry1* mRNA were significantly reduced by the cotreatment of GSK‐J4 and leptin in the hypothalamus of ob/ob mice. K) Immunoblotting assay indicating the phosphorylation of Ern1 and Stat3 after leptin and GSK‐J4 administration.

To examine the dependency of GSK‐J4 effects on leptin signaling, we selected db/db and ob/ob mice to perform the experiments. The db/db strain is deficient in the leptin receptor, whereas the ob/ob strain lacks functional leptin hormone. The administration of GSK‐J4 was sustained for 3 weeks (Figure S6A, Supporting Information), but it did not produce a body weight reduction compared to the vehicle group (**Figure** **5**A). There was no obvious difference in the weight of adipose tissue between the two groups (Figure 5B). The GSK‐J4 treatment also failed to affect food intake (Figure 5C), insulin, and leptin levels (Figure 5D). In fact, the GSK‐J4 treatment neither reduced the concentration of fasting blood glucose (Figure S6B, Supporting Information) nor improved glucose homeostasis, as demonstrated by OGTT and ITT assays, respectively (Figure S6C,D, Supporting Information). These data suggest that the leptin signaling pathway may be the underlying mechanism for the antiobesity function of GSK‐J4.

We reasoned that the administration of exogenous leptin to ob/ob mice with GSK‐J4 should create a stronger outcome than the administration of leptin with the vehicle control. We chose a low dose of leptin, which is minimally effective in the ob/ob mice, to investigate whether GSK‐J4 can also increase the sensitivity of ob/ob mice to extremely low doses of leptin (0.1 mg kg^−1^). We thus administered either vehicle or GSK‐J4 to ob/ob mice and subsequently administered an injection of saline or a bolus dose of leptin to each group (Figure S7A, Supporting Information). The body weight records showed that separate GSK‐J4 or leptin treatment did not significantly influence body weight, but cotreatment with GSK‐J4 and leptin significantly blocked body weight increases (Figure 5E) and resulted in less fat mass in the leptin‐deficient ob/ob mice (Figure 5F). The ob/ob mice that received GSK‐J4 plus leptin consumed less food than any of the other groups (Figure 5G). In the GSK‐J4‐ and leptin‐cotreated ob/ob mice, the insulin concentration was clearly decreased (Figure 5H), while blood leptin levels were not significantly changed. The cotreatment obviously improved glucose homeostasis, as shown in the ITT assay (Figure 5I) but did not reduce the fasting blood glucose concentration (Figure S7B,C, Supporting Information). In the hypothalamus tissues, mRNA levels of Cry1 were significantly reduced by GSK‐J4 treatment (Figure 5J). The levels of H3k27me3 and phosphorylated Stat3 were restored in the cotreatment group (Figure 5K). These data suggest that the Kdm6a inhibitor possesses biological activity as a leptin sensitizer.

**Figure 6:**
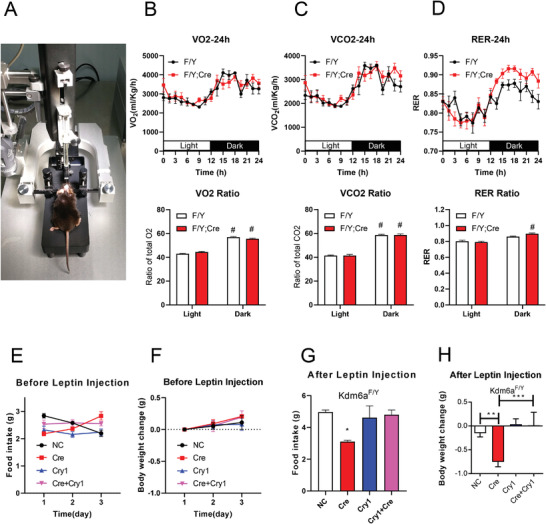
The specific *Kdm6a* knockout in the hypothalamus is responsible to leptin‐reduced the body weight and food intake. A) The mice received AAV of GFP or Cre microinjection in the hypothalamus (*n* = 6 in each group for (A)–(D)). B) Oxygen consumption (VO2) monitored over a 24 h period, shown as averaged values. #*p* < 0.05, light vs dark phage. C) Carbon dioxide production (VCO2) monitored over a 24 h period, shown as averaged values. #*p* < 0.05, light vs dark phage. D) The respiratory exchange rate (RER) was analyzed for 24 h. #*p* < 0.05, light vs dark phage. E) The mice received microinjection with AAV of GFP, Cre, Cry1, or Cre plus Cry1 in the hypothalamus (*n* = 6 in each group for (E)–(H)). Daily food intake before the leptin treatment was recorded. F) Body weight before the leptin treatment. G) Daily food intake after the mice received the leptin treatment. H) Body weight after the mice received the leptin treatment.

Next, we performed the microinjection of Cre‐expressing adeno‐associated virus (AAV) or control AAV into hypothalamus of Kdm6a^F/Y^ mice, followed by leptin treatment (**Figure** **6**A and Figure S8A, Supporting Information). As indicated in the Figure 6B–D, the rhythm of RER in Cre injection group has been changed that the ratio of RER during dark phage in Cre injection group was significantly higher than that of negative control group after the mice were treated with leptin. These data support the function of Kdm6a in the hypothalamus to mediate the leptin sensitivity. We next performed the stereotactic injection of AAV of vectors, Cre, Cry1, or Cre Plus Cry1 into hypothalamus of Kdm6a^F/Y^ mice (Figure S8B, Supporting Information). There were no significant changes of body weight or food intake before the mice received the leptin treatment (Figure 6E,F). However, the 24 h food intake of Cre injection group was significantly lower than other groups when the mice received the leptin injection (Figure 6G). Meanwhile, there were no significant differences of the food intake or body weight in Cre Plus Cry1 group when compared to the negative control group (Figure 6H). The bodyweight of Cre‐expressing AAV group are significantly lower than the Cre Plus Cry1 group, indicating that Cry1 functions as the key factor for Kdm6a‐mediated sensitivity to leptin in the hypothalamus.

### 2.4 KDM6A Expression in the Human Hypothalamus Correlates with ER Stress and Cry1

**Figure 7:**
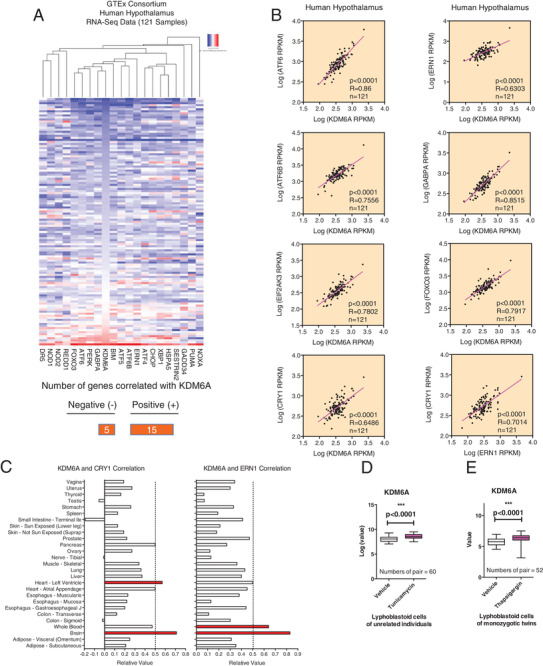
KDM6A expression in the human hypothalamus correlates with ER stress and CRY1. A) A correlation heatmap of KDM6A gene expression and unsupervised ER stress genes in 121 human hypothalamic tissue samples. A histogram of KDM6A gene expression levels is shown to the left of the heatmap. B) Representative scatter plots showing the correlation of KDM6A with the ATF6, ERN1, EIF2AK3, ATF6B, GABPA, FOXO3, and CRY1 genes. The correlation of ERN1 with the CRY1 was also examined. To determine the goodness of fit correlation (*R*), univariate linear regression was used. C) The KDM6A‐ERN1 and KDM6A‐CRY1 correlation was also calculated in different tissues. D) The relative expression of KDM6A in lymphoblastoid cells of unrelated individuals during tunicamycin challenge. E) The well‐known ER stress inducer thapsigargin also increased KDM6A mRNA levels in human lymphoblastoid cells.

**Figure 8:**
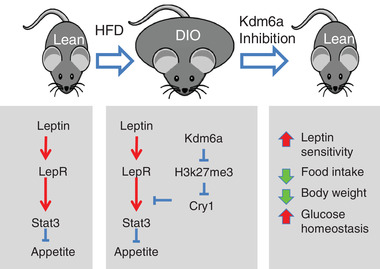
A schematic illustration of the proposed model in which Kdm6a inhibition sensitizes leptin signaling to reduce obesity‐related properties.

To determine whether the hypothalamic KDM6A and ER stress described above is relevant in humans, we utilized RNA‐seq data from 121 human hypothalamic tissues from the Genotype‐Tissue Expression (GTEx) project^[^ ^26^ ^]^ to calculate the correlations between *KDM6A* and the expression levels of ER stress‐associated genes (Table S2, Supporting Information). A correlation heatmap using unbiased hierarchical clustering revealed that ER stress‐associated genes were positively correlated with *KDM6A* expression (**Figure** **7**A). For example, as shown in the scatter plot, the expression of *ERN1, CRY1, EIF2AK3, ATF6, ATF6B, GABPA*, and *FOXO3* was positively correlated with *KDM6A* expression (Figure 7B). We also examined the correlations of *KDM6A*‐*ERN1* in different tissues of GTEx database and found that the most significant correlations were in the human brain and whole blood (Figure 7C and Table S3, Supporting Information). The highest correlation value between *KDM6A* and *CRY1* were found in brain and heart tissues. In previously published microarray data, we also found that both the ER stress inducer tunicamycin (Figure 7D) and thapsigargin (Figure 7E) significantly enhanced KDM6A mRNA levels. Together with our mouse data, these results suggest that Kdm6a inhibition sensitizes leptin signaling to reduce obesity‐related properties (**Figure** **8**).

## 3 Discussion

Obesity is characterized by hyperphagia and decreased energy consumption due to leptin resistance. Both the DIO mouse model and clinically obese patients exhibit very high blood concentrations of leptin. ER stress is deeply involved in leptin resistance. Many efforts have aimed to restore ER homeostasis and improve leptin resistance. However, limited data are available regarding the role of the H3k27me3 modification during ER stress stimulation. The H3k27me3 modification is specifically methylated by Ezh2 and demethylated by the Kdm6 family, which comprise Kdm6a and Kdm6b. In this study, we first induced ER stress with tunicamycin stimulation and validated that this treatment reduces the H3k27me3 modification via both the upregulation of Kdm6a and the downregulation of Ezh2. Furthermore, both *Kdm6a* knockout and pharmacological inhibition could reverse the decrease in H3k27me3 levels on target genes associated with ER stress. These findings encouraged us to examine the effect of Kdm6a inhibition in a mouse model of diet‐induced obesity.

Consistent with this notion, we found that Kdm6a deficiency was effective in hyperleptinemic and leptin‐resistant DIO mice and had minimal or no effect on lean, ob/ob, or db/db mice. Additional evidence revealing that the Kdm6a inhibitor can act as a leptin sensitizer comes from experiments in which we analyzed the physiological and biochemical responses to exogenous leptin administration. Our hypothesis concerning the ineffectiveness of GSK‐J4 in ob/ob mice was based on deficient leptin in the mouse model; exogenous administration of functional leptin in GSK‐J4‐pretreated ob/ob mice should have a greater effect than individual leptin treatment in ob/ob mice. Indeed, we found that when the GSK‐J4‐pretreated ob/ob mice were injected with leptin, food consumption and body weight were both significantly reduced relative to those in the vehicle‐pretreated mice. Although there are both deficiency of leptin signaling in ob/ob and db/bd mice, the reasons are different. The db/db mice are lack of functional leptin receptors, while the ob/ob mice are lack of functional leptin hormone. Therefore, only the ob/ob mice can respond the exogenous leptin injection. As for the increased leptin concentration by Kdm6a inhibitor, the leptin is mainly produced by adipocytes. The basal concentrations of leptin in the serum of db/db mice are much higher than that of ob/ob mice. Further examination should be performed to examine whether the Kdm6a inhibitor could stimulate the leptin production from the adipose of ob/ob mice.

We found that reduction of Cry1 through H3k27me3 modification by deactivation or removal of Kdm6a improves the properties of diet‐induced obesity. This is consistent with the published data that Cry1 KO mice are resistant to DIO. It is known that Cry1 mRNA exhibits robust circadian changes with nearly twofold changes.^[^ ^27^, ^28^ ^]^ And we also observed the most relevant correlation between KDM6A and CRY1 in the human hypothalamus, indicating that the KDM6A‐CRY1 interaction also exists in the human brain. However, the time of death also affects the Cry1 expression in post mortem human tissues. Further examination should be considered to examine the KDM6A‐CRY1 correlation more precisely in the human tissues.

Currently, there is no evidence that directly supports changes of Kdm6a in the hypothalamus reduce obesity phenotype. Both conditional KO and inhibitor treatment affects Kdm6a in any tissues in the body. We could not exclude the possibility that other mechanisms are involved in affecting the Cry1 expression. In site knock out the Kdm6a in the hypothalamus will greatly explain the role of Kdm6a. Actually, in Cry1^−/−^ mice, only partial resistance to HFD‐induced obesity was observed (≈18%),^[^ ^19^, ^22^ ^]^ while in the current study, a much greater effect was observed in body weight reduction following Kdm6a inhibition, which may suggest that Cry1 is not the only mediator and additional studies are needed to fully understand the underlying molecular mechanism.

The hypothalamus contains multiple nuclei important in homeostasis. Those include the master circadian pacemaker and other nuclei that regulate food intake, sleep, and core body temperature. These other nuclei have their own autonomous circadian oscillators of which the phase is antiphasic to the master circadian pacemaker. Cry1 is known to be rhythmic. Therefore, the timing of sampling and nuclei including the hypothalamus sample will affect Cry1 expression. We dissected the whole hypothalamus and these samples include most of nuclei of this part of brain. In fact, these methods are consistent to the previous studies, this procedure are easy to find the whole picture of the expression pattern.^[^ ^25^, ^29^ ^]^ However, in order to find the detailed Cry1 expression pattern, such as in which are the most important nuclei, further examinations with in situ hybridization or immunohistochemistry assays should be performed.

Notably, in most human populations, there is a strong association between obesity and acute lymphoblastic leukemia (ALL) leukemia, but the underlying mechanisms remain poorly understood.^[^ ^30^, ^31^, ^32^ ^]^ Patients with ALL have a high risk of obesity after treatment.^[^ ^33^, ^34^ ^]^ In addition, diet‐induced obesity can accelerate the development of ALL in mouse models.^[^ ^35^ ^]^ The leptin signaling pathway is also desensitized in ALL patients, even after the tumor has been cured, leading to the development of obesity following treatment. Importantly, the body mass index of ALL patients correlates with poor prognosis.^[^ ^36^ ^]^ Any attempt to improve leptin sensitivity will enhance the outcome of ALL patients and prevent recurrence, and the upregulation of leptin receptors by fasting can block the development of ALL.^[^ ^37^ ^]^ These data suggest that ALL treatments that promote resensitization to leptin might attenuate both obesity and ALL. The natural compounds withaferin A and celastrol belong to this class of leptin sensitizers^[^ ^25^, ^29^, ^38^ ^]^ and anti‐ALL agents.^[^ ^39^ ^]^ Our study thus supports GSK‐J4 as another candidate that possesses potential benefits for both obesity and ALL. As GSK‐J4 affects both Kdm6 members, further efforts should be performed to examine the contribution on Kdm6b in mediating the leptin sensitivity in the future study.

In summary, we found that the inhibition of Kdm6a is a promising leptin sensitization strategy for reducing food intake and body weight in obese mice. This study provides a new target for antiobesity strategies, expands the potential applications of Kdm6a inhibitors to combat obesity.

## 4 Experimental Section

### Cell Culture

MEFs were isolated from mice on postcoital day 12.5–13.5. Briefly, embryos were dissected into 10 mL of sterile phosphate buffer saline, voided of internal organs, and sheared through 18‐gauge syringes in the presence of 0.25% trypsin/1 × 10^−3^ m ethylene diamine tetraacetic acid. After a 15 min incubation with gentle shaking at 37 °C, dulbecco's modified eagle medium with 10% fetal bovine serum was added to inactivate trypsin. The cells were plated onto 100 mm cell culture dishes and incubated for 24 h at 37 °C. Adherent cells were used as MEFs. All MEFs in this study were used within five passages.

### Chemical Preparation

GSK‐J4 was purchased from Selleck (S7070), while tamoxifen (T5648) and glucose (G8270) were purchased from Sigma, insulin was purchased from Novo Nordisk (novolin R), and leptin was purchased from R&D Systems (498‐OB).

### Animals

The Kdm6a^F/Y^ and Kdm6a^F/F^ (Jackson Laboratory stock number 024177)^[^ ^40^ ^]^ and UBC‐Cre/ERT2 (Jackson Laboratory stock number 007179)^[^ ^41^ ^]^ mouse strains have been described previously. The mice were backcrossed with wild‐type C57BL/6J mice for at least ten generations. The mice were housed in a temperature‐controlled room (22 °C) with a 12 h light/dark cycle (light phage last from 8 a.m. to 8 p.m.; dark phage last from 8 p.m. to 8 a.m.) and given free access to food and water. The primers used for genotyping are listed in Table S5 in the Supporting Information. Ablation of the Kdm6a allele in vivo using the Cre/ERT2‐recombinase was achieved by daily i.p. injection of tamoxifen dissolved in corn oil (150 µL at 10 µg µL^−1^ to achieve a dose of 75 mg kg^−1^) over four consecutive days. For pharmacological inhibition of Kdm6a demethylase activity in vivo, GSK‐J4 dissolved in 2% dimethyl sulfoxide and H_2_O (final concentration at 3 mg mL^−1^ to achieve a dose of 30 mg kg^−1^ in vivo) was administered by daily i.p. injection at 10 a.m. (Zeitgeber times (ZTs) 2 h) over the course of 21 d (with ZT 0 defined as lights on and ZT 12 as lights off).

Animal experiments were performed in accordance with the relevant ethical regulations of the Shanghai Ninth People's Hospital. The study was approved by the Animal Experimental Ethics Committee of Shanghai Ninth People's Hospital, affiliated with the Shanghai Jiao Tong University School of Medicine. To induce the DIO model, six‐week‐old male mice were fed with either an HFD (60 kcal % fat content; Research Diets Formula D12331; Research Diets, Inc., New Brunswick, NJ) or a standard chow diet (ND; 11 kcal % fat content; Research Diets Formula D12329; Research Diets, Inc.) for 16 weeks. Body weight was recorded at the second hour of light phage weekly throughout the study. The mice were sacrificed by cervical dislocation. Tissues were collected from each mouse, snap frozen in liquid nitrogen and stored at −80 °C. Eight‐ or nine‐week‐old ob/ob, db/db mice were maintained on a standard chow diet.

For the intracerebral injection, Kdm6a^F/Y^ mice were anesthetized and received bilateral stereotaxic injections of adeno associated virus expressing green fluorescent protein (GFP), Cre, or Cry1 into the hypothalamus (1.6 mm posterior to the bregma, ±0.3 mm lateral to the midline, and 6 mm below the surface of the skull). The injection volume of 2 µL was delivered (0.25 µL min^−1^) in two injections into the hypothalamus. The adeno associated virus was purchased from Hanbio, China. After the mice received the intracerebral injection for 10 d, the leptin (0.1 mg kg^−1^) were injected i.p. for 2 d.

### Metabolic Rate and Physical Activity

Food intake, metabolic consumption, and locomotor activity were determined for animals fed ad libitum using the comprehensive laboratory animal monitoring system (Columbus Instruments, USA) according to the manufacturer's instructions. After mice could acclimate to the cage for 24 h, carbon dioxide (VCO2) and oxygen uptake (VO2) were measured, RER were calculated for the following 24 h. Voluntary activity was monitored from the *x*‐axis beam breaks collected every hour.

### Microcomputed Tomography Analysis

The hepatic structure and fatty liver measurement were evaluated using microcomputed tomography (µCT40, Scanco Medical, Switzerland), according to the previously reported methods.

### Leptin Administration to ob/ob Mice

Eight‐week‐old male ob/ob mice were treated with either vehicle or GSK‐J4 (30 mg kg^−1^) for seven consecutive days. On the eighth day, each group of mice was divided into two subgroups and each subgroup was injected with either saline or leptin (0.1 mg kg^−1^), together with vehicle or GSK‐J4, for two more weeks. Therefore, there were four groups: vehicle plus saline, vehicle plus leptin, GSK‐J4 plus saline, and GSK‐J4 plus leptin. The food intake and body weight were recorded of each mouse twice per week.

### OGTT and ITT

For the OGTT, the mice were fasted for 15 h (starting at 6 p.m. and lasting until 9 a.m.) and glucose (1 g kg^−1^ for lean mice and mice with DIO; 0.5 g kg^−1^ for *ob/ob* and *db/db* mice) was administered per os (P.O.). Blood glucose levels were measured from the tail before oral administration and 15, 30, 60, 90, and 120 min after administration.

For the ITT, the mice were fasted for 6 h, starting at 8 a.m. and lasting until 2 p.m. Recombinant human insulin (1 IU kg^−1^ for lean mice and mice with DIO; 2 IU kg^−1^ for *ob/ob* and *db/db* mice) was administered i.p. Blood glucose levels were measured from the tail before insulin administration and 15, 30, 45, and 60 min after administration.

### Hormone and Metabolite Measurements in Mouse Serum

The corresponding enzyme linked immunosorbent assay (ELISA) or assay kits were utilized according to the manufacturers’ instructions to measure plasma leptin (R&D), insulin (Millipore), cholesterol (Roche), and triglycerides (Roche). 5 µL of serum sample was used from lean mice and 5 µL of 5 × diluted serum sample from mice with DIO and db/db mice for the leptin ELISA. 5 µL of serum was used from lean mice, mice with DIO and ob/ob or db/db mice for the insulin ELISA, 3 µL for the cholesterol assay, and 3 µL for the triglyceride assay.

### H&E Staining

At the end of the treatment period, the adipose and liver tissue were dissected and it was stored in 10% buffered formalin phosphate. Paraffin‐embedded sections were H&E stained.

### Chromatin Immunoprecipitation (ChIP)

MEFs were treated with or without tunicamycin (1 µg mL^−1^) for 6 h. ChIP assays were performed using an EZ ChIP kit (Cell Signaling Technology, 9003). Briefly, MEFs (1 × 10^7^ cells) were fixed with 1% formaldehyde and then neutralized by adding 0.125 m glycine. Cells were collected and lysed in cell lysis buffer containing sodium dodecyl sulfate (SDS) and a cocktail of protease inhibitors. The lysates were sonicated to obtain soluble chromatin with an average length of 500 bp. After a 1:10 dilution in dilution buffer, the chromatin solutions were precleared and incubated with IgG or anti‐H3k27me3 antibodies. Next, the mixtures were incubated overnight at 4 °C on a rotating platform. The immunocomplexes were captured by protein A/G‐Sepharose beads. After extensive washing, the bound DNA fragments were eluted, and the resulting DNA was subjected to real‐time PCR analysis using the ChIP primer pairs listed in the Table S5 in the Supporting Information.

### ChIP‐Seq by Illumina HiSeq

ChIP samples were quantified using a Qubit 2.0 Fluorometer (Invitrogen, Carlsbad, CA, USA) and qualified by Agilent Bioanalyzer 2100 (Agilent Technologies, Palo Alto, CA, USA). Next generation sequencing library preparations were constructed following the manufacturer's protocol (NEBNext UltraII DNA Library Prep Kit for Illumina). For each sample, At least 10 ng ChIP product was used for library preparation. The ChIP product was treated with End Prep Enzyme Mix for end repairing, 5’ Phosphorylation and dA‐tailing in one reaction, followed by ligation to adaptors with a “T” base overhang. Adaptor‐ligated DNA was then recovered using AxyPrep Mag PCR Clean‐up (Axygen). Each sample was then amplified by PCR for eight cycles using P5 and P7 primers, with both primers carrying sequences which can anneal with flowcell to perform bridge PCR and P7 primer carrying a six‐base index allowing for multiplexing. The PCR products were cleaned up using AxyPrep Mag PCR Clean‐up, validated using an Agilent 2100 Bioanalyzer, and quantified by Qubit 2.0 Fluorometer. Then libraries with different indexes were multiplexed and loaded on an Illumina HiSeq instrument according to manufacturer's instructions (Illumina, San Diego, CA, USA). Sequencing was carried out using a 2 × 150 paired‐end configuration; image analysis and base calling were conducted by the HiSeq Control Software + offline basecaller + GAPipeline‐1.6 (Illumina) on the HiSeq instrument. The sequences were processed and analyzed by GENEWIZ. The data that support the findings of this study have been deposited in gene expression omnibus (GEO) with the accession codes GSE131068.

### Immunoblotting Analysis

The cells were lysed in lysate solution, and the proteins were separated on 8, 10, or 12% SDS‐polyacrylamide gel electrophoresis gels and transferred to polyvinylidene fluoride membranes. Next, 5% milk powder‐containing buffer was used to reduce the nonspecific background. Bands were detected using various antibodies as indicated. The membranes were incubated with primary antibodies at 4 °C overnight and secondary antibodies for 2 h at room temperature before exposure to an AI600 system in the dark for band detection. The catalog numbers of the primary antibodies are listed in Table S4 in the Supporting Information.

### mRNA Microarray Analysis

The hypothalamic tissues were collected at ZT 6 h. The mRNA expression profiles were investigated using the Agilent SurePrint G3 Mouse GE V2.0 array 8 × 60 K (Shanghai OE Biotech, China). The data that support the findings of this study have been deposited in GEO with the accession codes GSE115113. The RNA samples were first reverse transcribed into cDNA and these cDNA samples were then labeled using a Low Input Quick‐Amp Labeling Kit (Agilent Technologies, Santa Clara, CA, USA). Labeled cDNA samples were used as probes to hybridize to mRNA microarrays. After the samples were hybridized, the microarrays were scanned with an Agilent microarray scanner. Feature Extraction software (version 10.7.1.1, Agilent Technologies) was used to analyze array images to obtain raw data. GeneSpring software (version 14.8, Agilent Technologies) was employed to finish the basic analysis of the raw data. Initially, the raw data were normalized using the quantile algorithm. The probes with at least 100% of samples in any one condition out of two conditions having flags that indicate “Detected” were chosen for further data analysis. Differentially expressed mRNAs were then identified through fold change, and *p* values were calculated using *t*‐tests. The thresholds set for up‐ and downregulated genes were a fold change ≥ 1.5 and a *p*‐value ≤ 0.05.

### Reverse‐Transcription Polymerase Chain Reaction and Quantitative PCR Assays

The quantitative PCR was performed using an ABI Prism 7300 system (Applied Biosystems, USA) and SYBR Green (Takara, Dalian, China). For PCR, up to 1 µL of cDNA was used as a template. The thermal cycling conditions were 95 °C for 10 s followed by 40 cycles of 95 °C for 5 s and 60 °C for 30 s. A primer efficiency of >90% was confirmed with a standard curve spanning four orders of magnitude. Following the reactions, the raw data were exported using 7300 System Software 4 v1.3.0 (Applied Biosystems) and analyzed. The primers used are listed in Table S5 in the Supporting Information.

### Statistical Analysis

Figures were produced using GraphPad prism software. Animals were randomized based on blood glucose. Based on extensive experience with the mouse models of obesity and diabetes (for example, assay sensitivity, the different animal strains used, mortality rate) and given the planned analytical framework, the number of mice per group that would be required to detect effects of interest at the *p* < 0.05 level of significance was estimated. The numbers of technical replicates or biological replicates (independent experiments for cell culture, or individual mouse for in vivo) in each group were stated in the figure legends. All values are means ± s.e.m. in bar graphs. For box plots, centerlines represent the median; limits represent quartiles; whiskers represent minimum and maximum values. Unpaired Student's *t*‐tests with no assumption of equal variance were used for comparisons between two groups. To determine goodness of fit correlation (*R*), univariate linear regression was used. For comparisons of more than two groups, Analysis of Variance (by general linear model) was used. When overall *F* test were significant (*p* < 0.05), post hoc comparisons using Tukey's method of adjustment were conducted to determine the location of any significant pairwise differences. Analysis was performed using GraphPad prism software (La Jolla, CA). A two‐sided *p* < 0.05 was considered statistically significant. The mice were excluded if they died before the end of the designated study period.

## Conflict of Interest

The authors declare no conflict of interest.

## Supporting information

Supporting Information
