# Supplementary material for: Restoration of H3k27me3 Modification Epigenetically Silences Cry1 Expression and Sensitizes Leptin Signaling to Reduce Obesity‐Related Properties
Source: Adv Sci (Weinh). 2021 May 13;8(14):2004319. doi: 10.1002/advs.202004319 (PMC8292908; doi:10.1002/advs.202004319)
Supplement: Supplementary file 1 — Supporting Information [file ADVS-8-2004319-s001.pdf]

© 2020 Wiley-VCH GmbH

ADVANCED SCIENCE
Open Access

Supporting Information

for *Adv. Sci.*, DOI: 10.1002/advs.202004319

Restoration of H3k27me3 modification epigenetically silences Cry1 expression and sensitizes leptin signaling to reduce obesity-related properties

Yan Wei[1,#], Jun Chen[2,3,#], Xing Xu[2,3,#], Fan Li[2,3], Kun Wu[2,3], Yingying Jiang[2,3], Yuqing Rao[4], Chen Zhao[1], Wantao Chen[2,3,], Xu Wang[2,3,*]*

Supplementary Materials

Supplementary Introduction

Supplementary Results

Supplementary Methods and materials

Supplementary Figure 1. H3k27me3 modifications are significantly reduced during tunicamycin-induced ER stress in MEFs.

Supplementary Figure 2. The differentially expressed genes were enriched by using terms from the KEGG pathway.

Supplementary Figure 4. Inducible knockout of Kdm6a reduces the body weight and food intake of DIO mice but not lean mice.

Supplementary Figure 5. The Kdm6a inhibitor GSK J4 reduces the body weight and food intake of DIO mice but not lean mice.

Supplementary Figure 3. Significantly 1.5-fold changed genes in circadian entrainment pathway enriched in KEGG database.

Supplementary Figure 6. The Kdm6a inhibitor GSK J4 cannot reduce the body weight and food intake of db/db mice.

Supplementary Figure 7. The Kdm6a inhibitor GSK J4 cannot reduce the body weight and food intake of ob/ob mice.

Supplementary Figure 8. The stereotactic injection of AAV into hypothalamus of Kdm6a$^{F/Y}$ mice.

Supplementary Table 1. Serum data from HFD, ND, db/db, ob/ob mice.

Supplementary Table 2. Expression profile of ER stress genes correlated with KDM6A in the Hypothalamus of GTEx Consortium.

Supplementary Table 3. Correlation value between KDM6A and ER stress genes.

Supplementary Table 4. Primary antibody list.

Supplementary Table 5. Oligonucleotide primer list.

Supplementary Table 6. Target sequence list of RNA interference.

Supplementary Intruduction

The perturbations of ER homeostasis that are triggered predominantly by the accumulation of unfolded proteins lead to the development of a condition referred to as ER stress, which subsequently activates a complex signaling cascade called the unfolded protein response (UPR)[18]. The UPR has three integrated aims: to initially restore normal cell function by halting protein translation, to degrade misfolded proteins, and to activate the signaling pathways that lead to increased production of molecular chaperones involved in protein folding, such as heat shock protein a5 (Hspa5)[19]. If these objectives are not achieved within a certain time window or if the distribution is prolonged, the UPR proceeds towards apoptosis to prevent further damage to the body. There are three main signaling pathways involved in the UPR, including endoplasmic reticulum to nucleus signaling 1(Ern1), eukaryotic translation initiation factor 2 alpha kinase (Eif2ak3) and activating transcription factor 6 (Atf6). Ern1 can activate the transcription factor Xbox binding protein 1 (Xbp1)[20]. Importantly, ER stress is tightly linked to the pathophysiology of several metabolic diseases[21], including obesity. Previous observations revealed that increased ER stress in the brain plays a central role in the development of leptin resistance and consequently, obesity[22]. It has been reported the nature product celastrol to reduce ER stress dramatically improves leptin sensitivity and reverse obesity[23]. More recently, a paper from the same group further reported that IL1R is required in the celastrol-induced anti-obese effect[24]. Progress in epigenetics has gradually revealed the important role of ER stress in regulating bodily functions. However, the physiological influence of Kdm6a-mediated demethylation on H3k27me3 during ER stress remains largely elusive.

Supplementary Results

An ER stress inducer decreases the H3k27 trimethylation (H3k27me3) modification in mouse embryo fibroblasts (MEFs)

To examine the effect of ER stress on histone modification, MEFs were treated with tunicamycin for 6 h. We noticed a gradual decrease in the H3k27me3

modification and a slightly reduction of H3k4me3 during ER stress (Supplementary Figure 1A and Supplementary Figure 2A). We performed the ChIP-Seq assay to examine how many genes H3k27me3 and H3k4me3 modifications significantly influenced. In line with the roles of H3k27me3 in the regulation of gene transcriptions, annotation of the peak in genomic positions to the closest genes indicated that many peaks were positioned after the transcription start site (TSS). The tunicamycin treatment significantly attenuated H3k27me3 modification in the mouse genome (Supplementary Figure 1B). When compared to the 23,008 H3k27me3 peaks of Vehicle group, the number of unique peaks under tunicamycin is 16,403 (Supplementary Figure 1C). The differentially expressed genes were enriched by using terms from the KEGG pathway (Supplementary Figure 2H and I).

Next, we examined how the H3k27me3 modification was decreased. According to the immunoblotting assays, the protein level of the H3k27me3-specific methyltransferase Ezh2 was significantly decreased in a dose-dependent manner (Supplementary Figure 1A and Supplementary Figure 2A). However, we observed different changes in Kdm6a and Kdm6b, both of which specifically demethylate the H3k27me3 modification. The protein levels of Kdm6a were moderately increased, while those of Kdm6b were downregulated (Supplementary Figure 1A and Supplementary Figure 2A). Moreover, the tunicamycin treatment stimulated the upregulation of *Kdm6a* mRNA and, to a lesser extent, *Kdm6b*, as well as the upregulation of various ER stress target genes, including *Ern1*, *Eif2ak3* and *Atf6*. However, the mRNA levels of *Ezh2* were not obviously changed (Supplementary Figure 1D). These data indicated that Kdm6b and Ezh2 levels were decreased, likely via posttranslational modification during ER stress.

As there are three major signaling pathways associated with ER stress, we next examined the possible pathways that mediate tunicamycin-induced *Kdm6a* mRNA elevation. After siRNA-mediated knockdown of endogenous *Ern1*, *Eif2ak3* or *ATF6* in MEFs, we observed that the si-*Ern1*-treated MEFs possessed the lowest Kdm6a mRNA levels during tunicamycin challenge for 6 h (Supplementary Figure 1E). Furthermore, Kdm6a protein levels were also markedly decreased after si-*Ern1*

treatment in MEFs (Supplementary Figure 1F and Supplementary Figure 2B and H). On the other hand, ectopic expression of Ern1 in MEFs led to elevated mRNA and protein levels of Kdm6a (Supplementary Figure 1G-H and Supplementary Figure 2C). These data indicate that Ern1 likely functions as an upstream regulatory factor that can transcriptionally activate Kdm6a expression during ER stress.

To further examine the correlation between H3k27me3 modification and ER stress gene expression, we performed immunoblotting assays. Consistent with a critical role of Kdm6a in this process, *Kdm6a*-deficient or GSK-J4-treated MEFs were relatively more resistant to tunicamycin-induced decreases in total H3k27me3 levels (Supplementary Figure 1I-J and Supplementary Figure 2D-E), leading to reduced *Ern1* and *Eif2ak3* expression (Supplementary Figure 1K). These data suggest that inhibiting Kdm6a could restore H3k27me3 modification and compromise ER stress. Notably, treatment with GSK-J4 restored the phosphorylation of Stat3 after leptin stimulation (Supplementary Figure 1L and Supplementary Figure 2F-G). Taken together, these results suggest that H3k27me3-mediated silencing is globally restored upon Kdm6a inhibition.

We found that ER stress induced nearly 4-fold expression of Kdm6a, which was reduced by half by siErn1. The siAtf6/Eif2ak3 also exhibited an effect. Therefore, even Ern1 may be dominant, Atf6/Eif2ak3 shall contribute as well. Of note, such experiments were performed in MEF but not in the mouse hypothalamic neurons. Further examination should be performed in the brain to identify whether Ern1, Atf6 or Eif2ak3 mediate the Kdm6a up regulation.

Supplementary Methods and materials

RNA interference

Two small RNA fragments for each gene and one negative control (a scrambled sequence) were synthesized by Ribobio Company, China. The target sequences to each gene were listed in the Supplementary Table 6. MEFs were transfected with different siRNAs using Lipofectamine 2000 (Life Technologies). Realtime PCR analysis was used to confirm the knockdown effect 24 h later.

Supplementary Figure 1

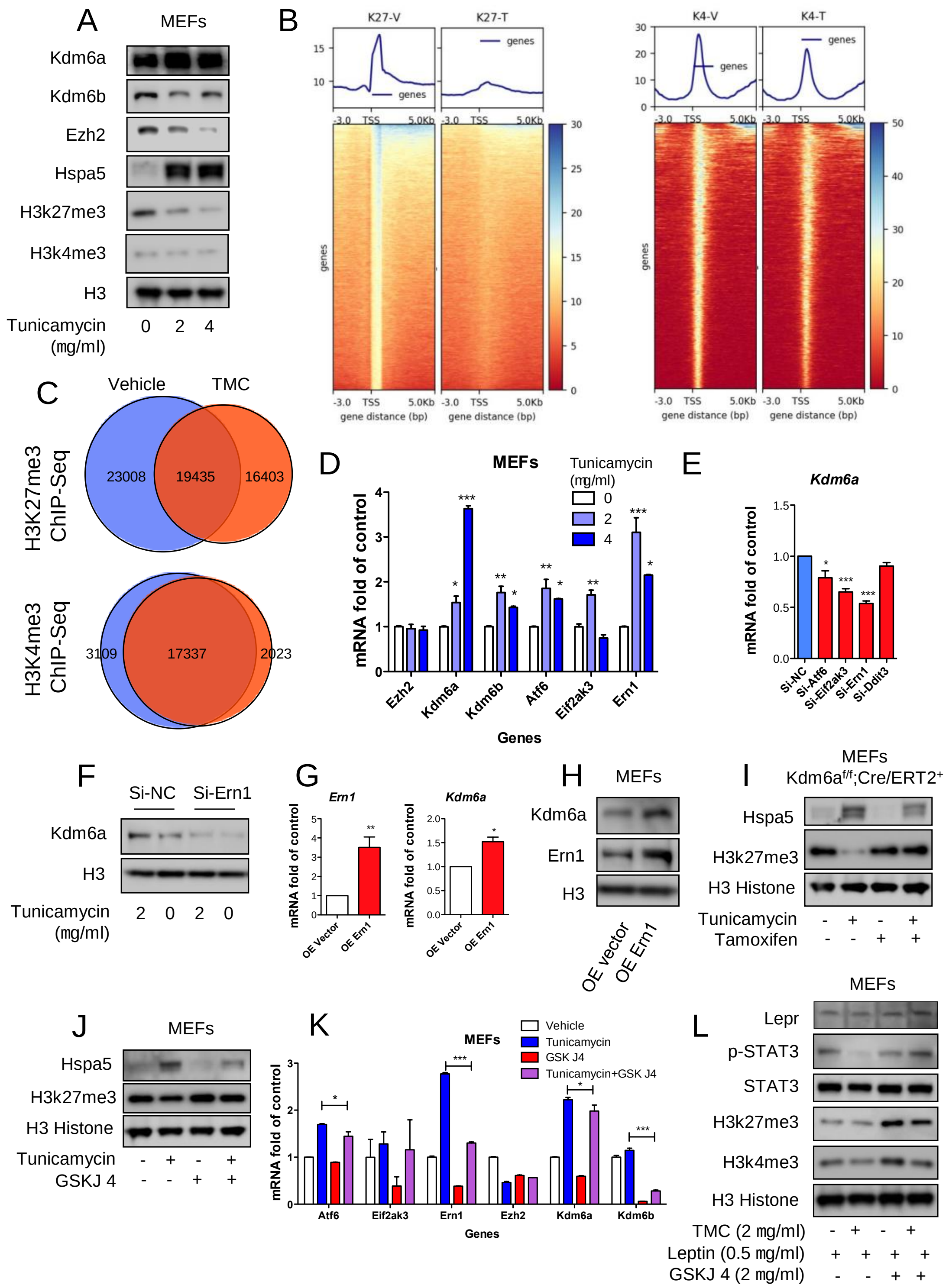

Supplementary Figure 1. H3k27me3 modifications are significantly reduced during tunicamycin-induced ER stress in MEFs.

A. Ezh2, Kdm6b and H3k27me3 levels were reduced, while Hspa5 and Kdm6a levels were increased in tunicamycin-treated MEFs. The quantified data were shown in the Supplementary Figure 2.

B. Heatmaps of H3k27me3 and H3k4me3 ChIP-Seq signals at TSS in Vehicle and tunicamycin treated MEFs.

C. Venn diagram showing the numbers of genes harboring H3k27me3 or H3k4me3 peaks in Vehicle and tunicamycin treated MEFs.

D. Tunicamycin induced *Kdm6a* and *Ern1* mRNA expression but not *Ezh2* mRNA expression in MEFs after 6 h of treatment. (n=3)

E. Real-time PCR assay showing that the depletion of *Ern1* by siRNA retarded tunicamycin treatment-induced *Kdm6a* mRNA induction in MEFs. (n=3)

F. The protein levels of Kdm6a were decreased in *Ern1*-specific siRNA-treated MEFs, even after tunicamycin stimulation.

G. Real-time PCR assay indicating that *Kdm6a* mRNA levels were also increased upon ectopic expression of Ern1 in MEFs. (n=3)

H. The protein levels of Kdm6a were increased upon ectopic expression of Ern1 in MEFs.

I. In the $Kdm6a^{F/Y}$;$Cre/ERT2^{+}$ MEFs, the tamoxifen-induced loss of *Kdm6a* retarded the reduction of H3k27me3 after tunicamycin treatment for 6 h.

J. The Kdm6a inhibitor GSK-J4 reversed the reduction of H3k27me3 after tunicamycin treatment for 6 h.

K. GSK-J4 treatment reduced *Ern1*, *Eif2ak3* and *Atf6* mRNA levels in the tunicamycin treated MEFs. All values in bar graphs represent the mean±s.e.m., n=3.

L. Immunoblotting assay indicating that GSK-J4 restored the phosphorylation of Stat3 and H3k27me3 during ER stress.

Supplementary Figure 2

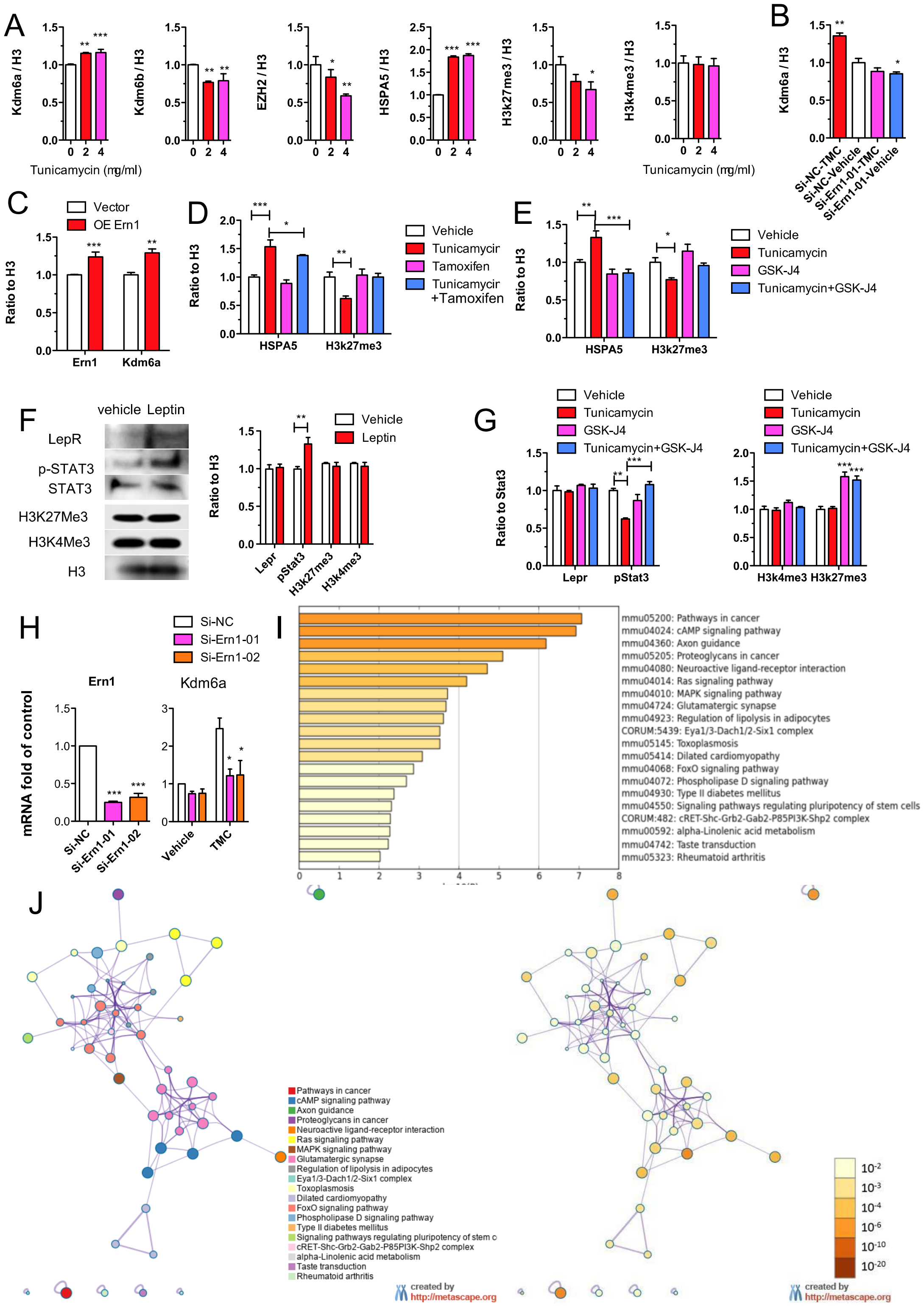

Supplementary Figure 2. The differentially expressed genes were enriched by using terms from the KEGG pathway.

A. The quantity data of Supplementary Figure 1A. *$p<0.05$, **$p<0.01$, ***$p<0.001$ compared to control group, n=3 for A-H.

B. The quantity data of Supplementary Figure 1F.

C. The quantity data of Supplementary Figure 1H.

D. The quantity data of Supplementary Figure 1I.

E. The quantity data of Supplementary Figure 1J.

F. The immunoblotting image of MEFs treated with vehicle or leptin, as well as the quantity data

G. The quantity data of Supplementary Figure 1L.

H. Realtime PCR for the RNAi specificity and efficiency of two si-Ern1 RNAs in MEFs

I. KEGG enrichment of signal pathway in the ChIP-Seq assay with H3k27me3 antibody.

J. The enrichment of protein-protein interaction based on the ChIP-Seq data.

Supplementary Figure 3

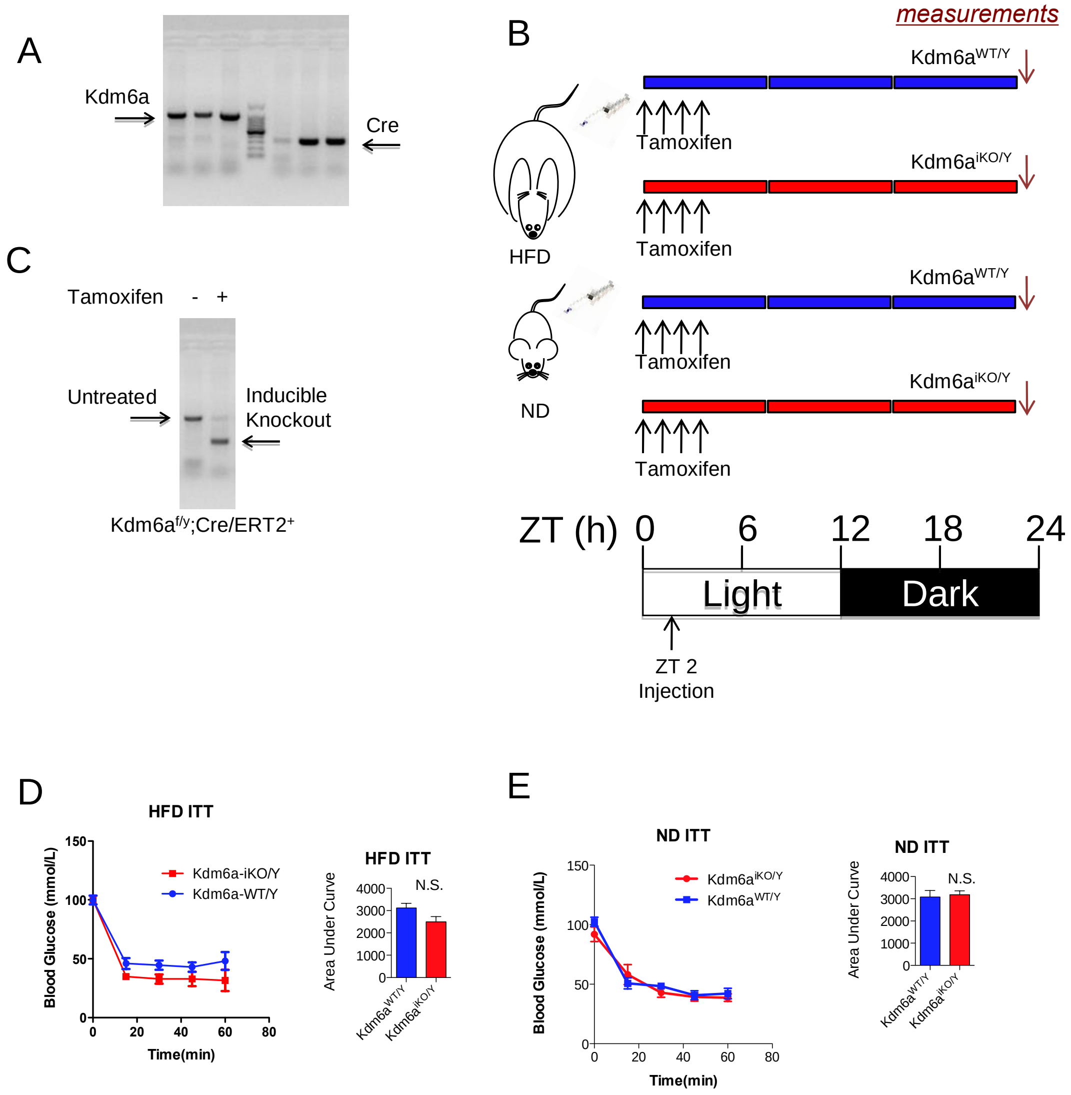

Supplementary Figure 3. Inducible knockout of Kdm6a reduces the body weight and food intake of DIO mice but not lean mice.

A. Establishment of $Kdm6a^{iKO/Y}$ mice.

B. A schematic illustration of Kdm6a inducible knockout.

C. Validation of Kdm6a inducible knockout with PCR assays.

D. The ITT assay to examine the sensitivity to insulin in DIO mice.

E. The ITT assay to examine the sensitivity to insulin in normal diet mice.

Supplementary Figure 4

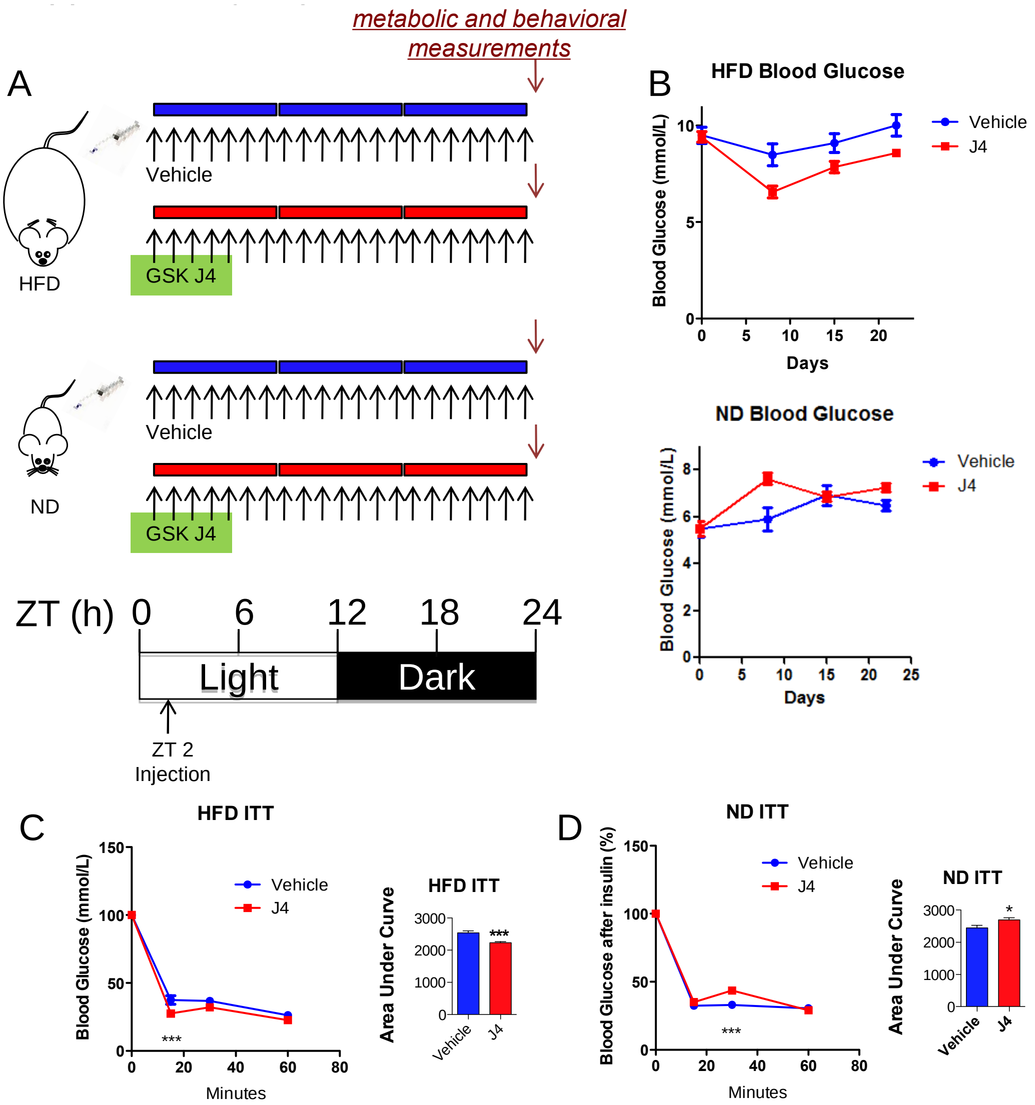

Supplementary Figure 4. The Kdm6a inhibitor GSK J4 reduces the body weight and food intake of DIO mice but not lean mice.

A. A schematic illustration of vehicle or GSK J4 administration during 3 weeks on mice.

B. The blood glucose of HFD or ND mice was recorded at indicated time points.

C. The ITT assay to examine the sensitivity to insulin in DIO mice.

D. The ITT assay to examine the sensitivity to insulin in normal diet mice.

Supplementary Figure 5

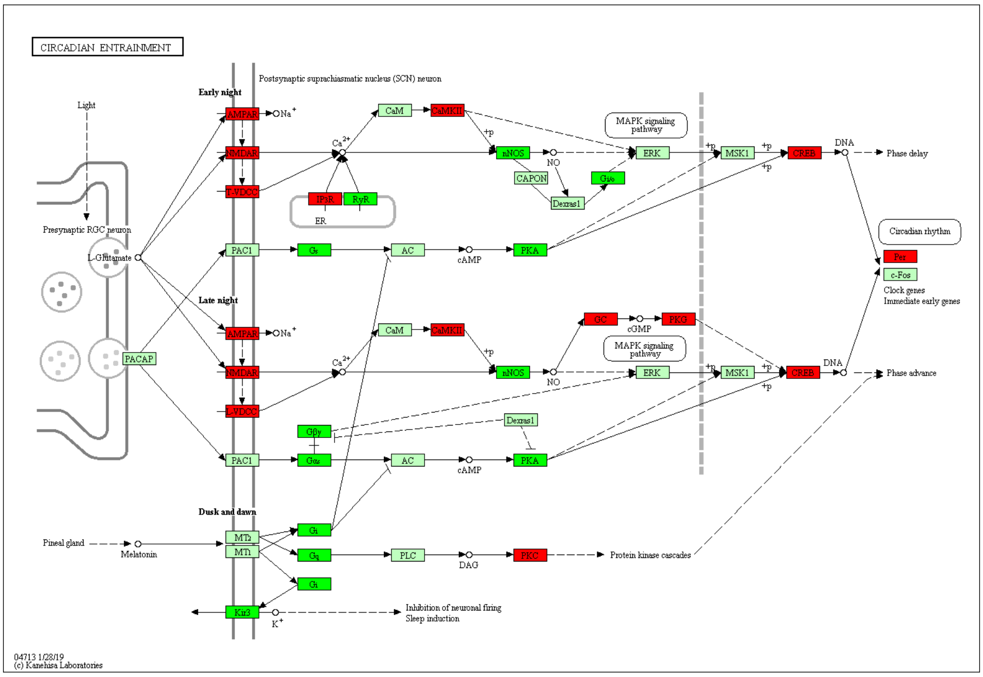

Supplementary Figure 5. Significantly 1.5-fold changed genes in Circadian entrainment pathway enriched in KEGG database.

The hypothalamus tissues in vehicle or GSK-J4 treated DIO mice were performed gene array assays. Upregulation over 1.5 fold was label in red, and downregulation over 1.5 fold was label in Green

Supplementary Figure 6

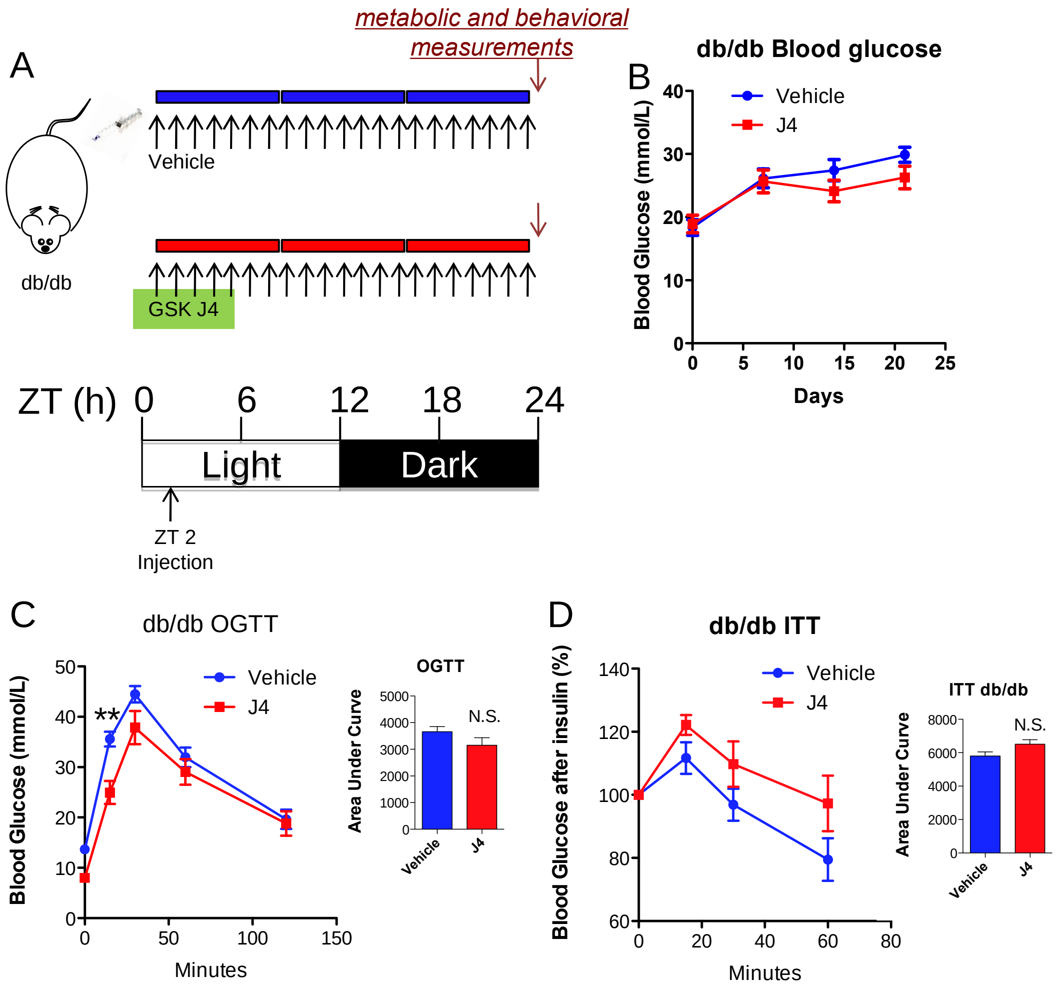

Supplementary Figure 6. The Kdm6a inhibitor GSK J4 cannot reduce the body weight and food intake of db/db mice.

A. A schematic illustration of vehicle or GSK J4 administration during 3 weeks on the db/db mice.

B. The blood glucose db/db mice were recorded at indicated time points.

C. The OGTT assay to detect the blood glucose homeostasis in the db/db mice.

D. The OGTT assay to detect the blood glucose homeostasis in the db/db mice.

Supplementary Figure 7

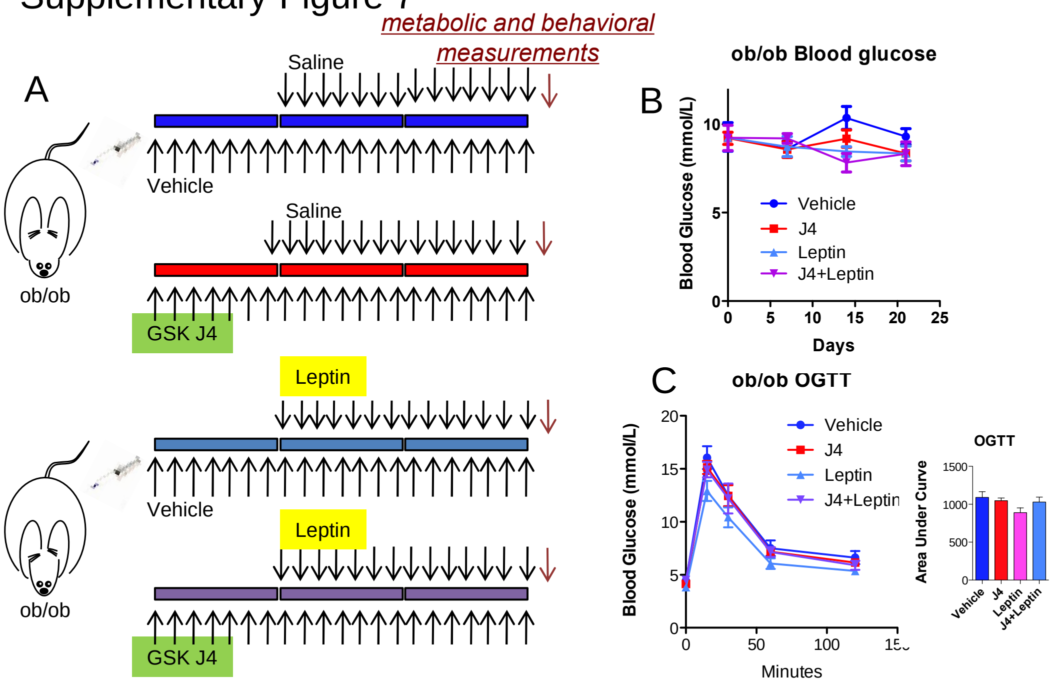

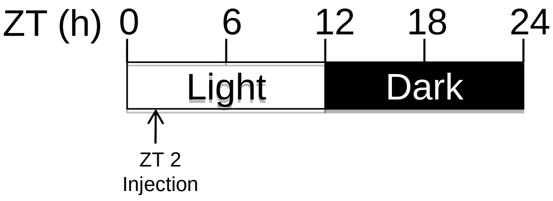

Supplementary Figure 7. The Kdm6a inhibitor GSK J4 cannot reduce the body weight and food intake of ob/ob mice.

A. A schematic illustration of vehicle or GSK J4 administration during 3 weeks on the ob/ob mice in the presence or absence of exogenous leptin administration.

B. The blood glucose ob/ob mice were recorded at the indicated time points.

C. The OGTT assay to detect the blood glucose homeostasis in the ob/ob mice.

Supplementary Figure 8

A

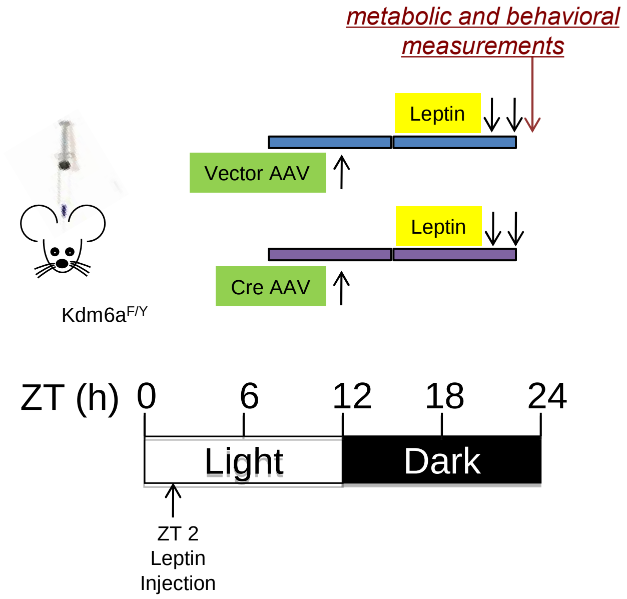

B

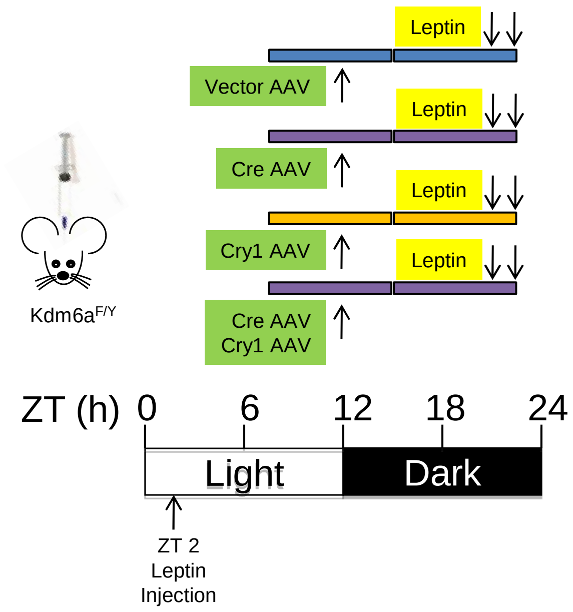

Supplementary Figure 8. The stereotactic injection of AAV into hypothalamus of Kdm6a$^{F/Y}$ mice.

A. A schematic illustration of stereotactic injection of AAV of vectors or Cre into hypothalamus of Kdm6a$^{F/Y}$ mice, followed by leptin injection and metabolic cage assay.

B. A schematic illustration of stereotactic injection of AAV of vectors, Cre, Cry1 or Cre Plus Cry1 into hypothalamus of Kdm6a$^{F/Y}$ mice followed by leptin injection.

Supplementary Table 1. Serum data from HFD, ND, db/db, ob/ob mice.

Mice	Group	Leptin	Insulin	TC (mmol/l)	TG (mmol/l)	HDL-c (mmol/l)	LDL-c (mmol/l)
Normal	Vehicle	1975.93±395.92	3.60±0.42	2.70±0.05	1.58±0.11	1.27±0.07	0.21±0.01
	GSK-J4	1524.79±211.86	4.25±0.44	2.51±0.09	1.06±0.05 ***	1.29±0.05	0.23±0.01
High Fat	Vehicle	40706.48±5802.07 ***	8.15±0.76 ***	4.47±0.20 ***	1.04±0.04 ***	1.71±0.08 ***	0.68±0.05 ***
	GSK-J4	19958.60±2783.06 ***	4.72±1.01 *	4.10±0.16	0.87±0.05 *	1.61±0.07	0.44±0.03 ***
db/db	Vehicle	7656.43±22.01	12.35±1.23	3.75±0.12	3.36±0.32	1.84±0.10	0.18±0.01
	GSK-J4	7665.6±21.46	12.61±1.21	3.84±0.2	2.33±0.33*	1.64±0.09	0.28±0.02***
ob/ob	Vehicle	260.38±9.04	21.16±0.38	4.30±0.09	1.65±0.07	1.88±0.05	0.31±0.01
	Vehicle+Leptin	248.02±20.77	19.69±0.52	4.76±0.08***	1.40±0.08*	1.90±0.15	0.34±0.01*
	GSK-J4	346.68±33.62	21.22±1.12	5.33±0.51	1.26±0.11**	2.22±0.12*	0.41±0.13
	GSK-J4+Leptin	236.03±12.07	17.3±1.1	4.43±0.11	1.32±0.07**	1.94±0.07	0.31±0.02

Data was expressed as Mean±SEM

**p*<0.05, ***p*<0.01, ****p*<0.005 vs Vehicle

Supplementary Table 2. Expression profile of ER stress genes correlated with KDM6A in the Hypothalamus of GTEx Consortium.

ER Stress gene	Pearson r	95% confidence interval	P value (two-tailed)	P value summary	Reference
ERN1	0.7638	0.6777 to 0.8293	< 0.0001	***	Nature. 2016 Apr 21;532(7599):394-7.
ATF6	0.9079	0.8705 to 0.9349	< 0.0001	***	Nature. 2016 Apr 21;532(7599):394-7.
ATF6B	0.8203	0.7518 to 0.8712	< 0.0001	***	
PERK	0.8251	0.7583 to 0.8748	< 0.0001	***	Nature. 2016 Apr 21;532(7599):394-7.
HSPA5	0.6415	0.5228 to 0.7357	< 0.0001	***	Nature. 2016 Apr 21;532(7599):394-7.
CHOP	0.4784	0.3279 to 0.6053	< 0.0001	***	Nature. 2016 Apr 21;532(7599):394-7.
XBP1	0.6179	0.4939 to 0.7173	< 0.0001	***	Nature. 2016 Apr 21;532(7599):394-7.
NOD1	0.5821	0.4504 to 0.6890	< 0.0001	***	Nature. 2016 Apr 21;532(7599):394-7.
NOD2	0.4635	0.3107 to 0.5930	< 0.0001	***	Nature. 2016 Apr 21;532(7599):394-7.
ATF4	0.6161	0.4917 to 0.7159	< 0.0001	***	Nature. 2016 Apr 21;532(7599):394-7.
ATF5	0.7657	0.6801 to 0.8307	< 0.0001	***	F1000Research 2017, 6(F1000 Faculty Rev):1897
GADD34	0.2533	0.07832 to 0.4132	0.0051	**	F1000Research 2017, 6(F1000 Faculty Rev):1897
BIM	0.8714	0.8205 to 0.9086	< 0.0001	***	F1000Research 2017, 6(F1000 Faculty Rev):1897
PUMA	0.5707	0.4366 to 0.6800	< 0.0001	***	F1000Research 2017, 6(F1000 Faculty Rev):1897
DR5	0.6066	0.4800 to 0.7084	< 0.0001	***	F1000Research 2017, 6(F1000 Faculty Rev):1897
NOXA	0.5681	0.4335 to 0.6779	< 0.0001	***	F1000Research 2017, 6(F1000 Faculty Rev):1897
REDD1	0.2702	0.09630 to 0.4281	0.0027	**	F1000Research 2017, 6(F1000 Faculty Rev):1897
SESTRIN2	0.3296	0.1605 to 0.4799	0.0002	***	F1000Research 2017, 6(F1000 Faculty Rev):1897
GABPA	0.915	0.8803 to 0.9400	< 0.0001	***	F1000Research 2017, 6(F1000 Faculty Rev):1897
FOXO3	0.8349	0.7714 to 0.8820	< 0.0001	***	Cell Metab. 2014 Nov 4;20(5):870-81.

Supplementary Table 3. Correlation value between KDM6A and ERN1 in GTEx.

Tissue	KDM6A-ERN1 R value	KDM6A-CRY1 R value
Adipose – Subcutaneous	0.35	0.21
Adipose – Visceral (Omentum)	0.31	0.25
Brain	0.83	0.71
Whole Blood	0.64	0.47
Colon – Sigmoid	0.3	-0.019
Colon – Transverse	0.11	0.12
Esophagus – Gastroesophageal Junction	0.44	0.2
Esophagus – Mucosa	0.13	0.22
Esophagus – Muscularis	0.38	0.23
Heart – Atrial Appendage	0.45	0.5
Heart – Left Ventricle	0.5	0.57
Liver	0.4	0.37
Lung	0.43	0.36
Muscle – Skeletal	0.36	0.34
Nerve – Tibial	0.13	-0.0088
Ovary	0.12	0.27
Pancreas	0.27	0.5
Prostate	0.47	0.22
Skin – Not Sun Exposed (Suprapubic)	0.18	0.19
Skin – Sun Exposed (Lower leg)	0.21	0.13
Small Intestine – Terminal Ileum	0.41	-0.19
Spleen	0.19	0.13
Stomach	0.33	0.26
Testis	0.07	-0.055
Thyroid	0.067	0.16
Uterus	0.3	0.27
Vagina	0.34	0.19

Supplementary Table 4. Primary antibody list.

Antigen	Company	Catalog Number
Kdm6a	Cell Signaling Technology	#33510
Kdm6b	Cell Signaling Technology	#3457
Ezh2	Cell Signaling Technology	#5246
H3k27me3	Cell Signaling Technology	#9733
H3k4me3	Cell Signaling Technology	#9751
H3 histone	Cell Signaling Technology	#4499
Hspa5	Cell Signaling Technology	#3177
Ern1	Cell Signaling Technology	#3294
p-Ern1	Abcam	ab48187
Eif2ak3	Cell Signaling Technology	#3192
Lepr	Abclonal	A2999
p-Stat3	Cell Signaling Technology	#9136
Stat3	Cell Signaling Technology	#9139

Supplementary Table 5. Oligonucleotide primer list.

Target Gene	Forward primer	Reverse primer
Kdm6a	CGGGCGGACAAAAGAAGAAC	CATAGACTTGCATCAGATCCTCC
Kdm6b	TGAAGAACGTCAAGTCCATTGTG	TCCCGCTGTACCTGACAGT
Ezh2	AGCACAAGTCATCCCGTTAAAG	AATTCTGTTGTAAGGGCGACC
Ern1	ACACTGCCTGAGACCTTGTTG	GGAGCCCGTCCTCTTGCTA
Eif2ak3	CGCGTCGGAGACAGTGTTT	GTCCTCCACGGTCACTTCG
Atf6	GAAGTCCGAGCCATCCTCTC	TGATCTGGACCGTTTCAAAGG
Atf6b	GAAGTCCGAGCCATCCTCTC	TGATCTGGACCGTTTCAAAGG
Hsp90b1	GTTCGTCAGAGCTGATGATGAA	GCGTTTAACCCATCCAACTGAAT
Ube2v1	CGGGAGTAAAAGTCCCTCGAA	GGAGGTCCAATTATCATGCCTGT
Grin2a	ACGTGACAGAACGCGAACTT	TCAGTGCGGTTCATCAATAACG
Grin2b	CAGCAAAGCTCGTTCCCAAAA	GTCAGTCTCGTTCATGGCTAC
Pld1	GCCGCAGATATGAGCAACCT	GGATGTTGGGCTCCTTAAATCCT
Gapdh	AATGGATTTGGACGCATTGGT	TTTGCACTGGTACGTGTTGAT
β-actin	GAGTCCTACGACATCATCGCT	CGTCCGACATAGTTTGGGAAA
Cry1	CACTGGTTCCGAAAGGGACTC	CTGAAGCAAAAATCGCCACCT
Clock	ATGGTGTTTACCGTAAGCTGTAG	CTCGCGTTACCAGGAAGCAT
Rora	GTGGAGACAAATCGTCAGGAAT	TGGTCCGATCAATCAAACAGTTC
Kdm6a genotyping	AACAAAAACCCAGGCTTTATTCAC	AGTTTCAGGATACCTTTACTATAAG
Cre genotyping	ATTTGCCTGCATTACCGGTCG	CAGCATTGCTGTCACTTGGTC

Supplementary Table 6. Target sequence list of RNA interference.

Target Gene	Target sequence
Si-Ern1-001	GCTAACGCCTACTCTGTAT
Si-Ern1-002	GCTCCATCAAGTGGACTTT
Si-ATF6	GGGCAGGACTATGAAGTAA
Si-Eif2ak3	CGGGAAAACGGTTCTGAGA
Si-Ddit3	AGCCTAACACGTCGATTATATCA
